# Improving the integrity and reproducibility of research that uses antibodies: a technical, data sharing, behavioral and policy challenge

**DOI:** 10.1080/19420862.2024.2323706

**Published:** 2024-03-06

**Authors:** M. Biddle, P. Stylianou, M. Rekas, A. Wright, J. Sousa, D. Ruddy, M. I. Stefana, K. Kmiecik, A. Bandrowski, R.A. Kahn, C. Laflamme, E. M. Krockow, H.S. Virk

**Affiliations:** a NIHR Respiratory BRC, Department of Respiratory Sciences, University of Leicester, Leicester, UK; b JDRF/Wellcome Diabetes and Inflammation Laboratory, Wellcome Centre for Human Genetics, Nuffield Department of Medicine, University of Oxford, Oxford, UK; c Department of Neuroscience, UC San Diego, La Jolla, CA, USA; d Department of Biochemistry, Emory University School of Medicine, Atlanta, USA; e Department of Neurology and Neurosurgery, Structural Genomics Consortium, The Montreal Neurological Institute, McGill University, Canada; f School of Psychology and Vision Sciences, University of Leicester, Leicester, UK

**Keywords:** Antibodies, antibody characterization, antibody validation, monoclonal antibodies, OGA (only good antibodies), open science, recombinant antibodies, reproducibility, RRID (research resource identification initiative), YCharOS (antibody characterization through open science)

## Abstract

Antibodies are one of the most important reagents used in biomedical and fundamental research, used to identify, and quantify proteins, contribute to knowledge of disease mechanisms, and validate drug targets. Yet many antibodies used in research do not recognize their intended target, or recognize additional molecules, compromising the integrity of research findings and leading to waste of resources, lack of reproducibility, failure of research projects, and delays in drug development. Researchers frequently use antibodies without confirming that they perform as intended in their application of interest. Here we argue that the determinants of end-user antibody choice and use are critical, and under-addressed, behavioral drivers of this problem. This interacts with the batch-to-batch variability of these biological reagents, and the paucity of available characterization data for most antibodies, making it more difficult for researchers to choose high quality reagents and perform necessary validation experiments. The open-science company YCharOS works with major antibody manufacturers and knockout cell line producers to characterize antibodies, identifying high-performing renewable antibodies for many targets in neuroscience. This shows the progress that can be made by stakeholders working together. However, their work so far applies to only a tiny fraction of available antibodies. Where characterization data exists, end-users need help to find and use it appropriately. While progress has been made in the context of technical solutions and antibody characterization, we argue that initiatives to make best practice behaviors by researchers more feasible, easy, and rewarding are needed. Global cooperation and coordination between multiple partners and stakeholders will be crucial to address the technical, policy, behavioral, and open data sharing challenges. We offer potential solutions by describing our Only Good Antibodies initiative, a community of researchers and partner organizations working toward the necessary change. We conclude with an open invitation for stakeholders, including researchers, to join our cause.

## Introduction

Antibodies are routinely used to detect, label, and quantify specific target molecules present in patient-derived samples in multiple different applications. In fact, antibodies are one of the most important tools used in biomedical research, where they play critical roles in the discovery and development of new drugs. However, since at least 2008, it has been clear that many research antibodies either do not recognize their target or are unselective, i.e., additionally label multiple unrelated targets.^1–6^ A suitable research antibody is one that binds the intended target selectively in the application of interest and is renewable. Recombinant antibodies are an exemplar renewable technology because they can be potentially infinitely regenerated with reduced lot-to-lot variation in comparison to older technologies. This last feature is central to the goal of reproducibility of data reported.

The use of nonselective antibodies and insufficient validation have contributed to the reproducibility crisis in biomedical research, hampered drug development, led to the failure of many research projects, and in some cases, led entire scientific fields in the wrong direction. Overall, this has resulted in a colossal waste of time, research funding, animals used in the production of antibodies, and research using those antibodies.^7–10^ Irreproducible research is thought to cost $28 billion per year, with approximately $350 million attributed to the use of bad antibodies in the US alone.^8,11^ Others have estimated >$1 billion is wasted on poorly performing antibodies annually.^12^ An issue that we believe may be under-appreciated is the direct impact that the use of poorly performing antibodies has on the research participants and patients who donate their tissues, blood, cells, and postmortem bodies in the expectation that they will support research; this problem means that their valuable donation may be wasted. Similarly, large numbers of animals are wasted in the production of nonselective antibodies, and in research using these antibodies.

**Figure 1.**
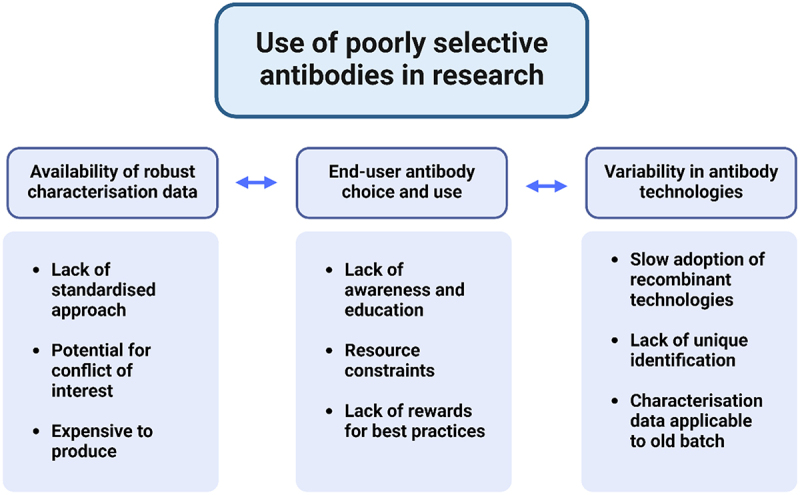
Determinants of the use of poorly selective antibodies in research.

The problem has been described as the “antibody horror show”,^1^ and the result of the problem has been described as “littering the literature with false findings”.^3^ We believe that the problem is driven by a number of factors within the research environment, where the users’ behaviors interact with inherent variability of some technologies, and a lack of robust quality control data and mechanisms (Figure 1).

The behavior of end-users is a key component, in part because inattention to the issue of reagent validation maintains a marketplace that includes a high proportion of non-selective antibodies. The factors that determine their capability, opportunity and motivation for best practices are a focus for this program. However, the issue is also maintained by a lack of efficient quality control mechanisms. Robust quality control experiments can be expensive and slow, and the number of antibodies used in research makes this difficult to address. End-users are also not aware of the necessity of quality controls and frequently do not perform this themselves, nor find and use robust data from others. Polyclonal antibody technologies, currently popular amongst end-users, have inherently high lot-to-lot variation, although newer recombinant antibodies have less variation, their adoption by end-users has been slow. These issues are discussed in more detail within the main text.

The establishment of a consensus position on antibody validation, known as the “5 pillars” (see below) and based on specific methods that should be used to validate antibodies, represents a notable step forward.^6^ The recommendations suggest that antibodies need to be validated in an application-specific manner because the antigen they recognize will change conformation between applications. For example, western blotting is usually performed on denatured samples, with the antigen taking an unfolded conformation, while in immunoprecipitation the antigen is in a more native folded conformation. The selectivity of an antibody will also be affected by the number of similar antigens present in the assay, which can vary substantially between assay, cell type, and tissue. Therefore, validation needs to be sample type and application specific. What may appear as minor differences in protocols for the same technique may also affect antibody performance. This can make robust antibody validation a real challenge. The five pillars are presented as alternative and complementary approaches for antibody validation with confidence gained with each pillar used for any antibody. Uhlen et al.^6^ do not present them as a hierarchy, but instead suggest at least one should be used, preferably more.

The consensus recommended **first pillar** is genetic strategies^6^ to use as optimal negative controls for antibody specificity. This ideally involves specifically removing the gene of interest, using CRISPR-Cas9 for example, to confirm that gene knockout removes antibody staining. The use of short interfering RNA (siRNA) or short hairpin RNA (shRNA) to reduce expression of a target (knockdown) is an alternative that can be used when complete removal of the gene affects cell viability or proliferation. The results of knockdown studies can be more difficult to interpret than knockout, as some signal will remain, and the expression of unrelated genes can also be altered downstream or through off-target effects of the knockdown. Genetic strategies are not feasible in some applications.

The **second pillar** is orthogonal strategies to compare antibody staining to protein/gene expression using an antibody-independent method, e.g., targeted mass spectroscopy.^6^ The proposal presents a method that includes multiple samples with varied protein expression. The orthogonal approach is frequently used, particularly for applications where genetic strategies are not feasible, such as immunohistochemistry on human tissue. Immunohistochemistry validation can be particularly challenging, as antigen conformation will be different between the wide variety of antigen retrieval methods used (e.g., boiling, high/low pH buffers). This can involve staining multiple tissues with varying RNA expression of the gene of interest and comparing to antibody staining intensity. A weakness of this approach is that RNA expression does not necessarily correlate strongly with protein expression.^13^ It should also be noted that several samples would be required to establish a statistically significant correlation between different approaches, and most vendors and publications do not include this calculation when presenting orthogonal validation. The proposal does not specifically address how many samples need to be used and whether the calculation of a coefficient is required.

The **third pillar** is independent antibodies, where the antibody reactivity is compared to another antibody that detects an entirely different epitope of the same target antigen in multiple samples with different levels of expression.^6^ In immunohistochemistry this can involve comparing the staining pattern (the distribution of antibody staining within a tissue and/or its subcellular localization) between independent antibodies. There can be an element of subjectivity in terms of defining a staining pattern, and the pattern may be similar for many proteins. Nonetheless, this approach is recommended because it can provide supportive evidence for the selectivity of an antibody. A challenge with this approach for commercial antibodies is that the exact epitope targeted is often not disclosed, making it difficult to know whether antibodies are truly independent.

The **fourth pillar** is tagged protein expression, which involves heterologous expression of the target with a tag, such as a fluorescent protein, FLAG or hemagglutinin (HA) epitope.^6^ The antibody staining can then be compared to the expression of the tag, which might be detected using different antibodies or directly in the case of fluorescent proteins. This method is only possible for certain applications where heterologous expression systems can be used. It is also necessary to consider that heterologous expression can result in very high expression of the target relative to endogenous levels, which may result in the antibody appearing more selective than in the final intended experimental application.

The **fifth pillar** and final recommended approach is immunocapture followed by mass spectroscopy.^6^ This approach, which relies on the peptide sequencing of proteins that were captured by an antibody, is useful for any experimental method that relies on immunocapture. The sequenced peptides, however, will include both antigens directly captured by the antibody, and those that interact with the captured antigen. With this method it can be difficult to establish whether the identification of peptides from other proteins represents an interaction partner of the target protein, or the off-target binding of an antibody. The recommendations suggest that the top three peptide sequences all coming from the target of interest would constitute good evidence of antibody selectivity.

Establishment of these consensus recommendations, despite the caveats described above, represent a major step forward. However, our recent analysis shows that data conforming to these recommendations is rarely presented in the literature.^14^ The underlying reasons for this are complex and discussed in more detail below, but clearly end-users of antibodies are not currently performing validation to the required standard to ensure the integrity and reproducibility of research findings.

In this opinion article we describe some of the key initiatives and approaches around large-scale antibody validation/characterization, community data sharing, researcher education, and development of data standards that aim to address this problem, and the progress they have made and are making. We briefly describe our view of the open data generated by YCharOS, as one of the initiatives in this area, and its relation to other initiatives. We then describe the remaining challenges and outline some of the ongoing work, including by the OGA community, to address these.

## Progress so far

### Behavioural solutions

To our knowledge, very little research has focused on providing insights into the psychosocial factors that shape end-user behavior in the context of antibodies. Using a survey devised with the Global Biological Standards Institute, Freedman et al.^15^ aggregated responses from over 500 researchers of different career levels to show that a higher researcher experience level was associated with better self-reported validation behavior. Researchers reported that the main barriers to validation were the time it takes and/or delays it introduces, followed by cost, followed by not believing it was necessary. The authors’ main conclusions drawn from this survey were that education and training of junior researchers was a key priority, and that developing a more rapid and less expensive validation methodology would help eliminate the problem.

As will be detailed in a future report, our focus groups have also identified that individual researchers feel the necessary validation work is not supported by the reward structures of science, and some felt it should not be their responsibility. Researchers also highlighted that it is possible to fund, approve and publish research using unsuitable antibodies. They further expressed a need for additional support to find and use the best available data to inform their antibody purchasing and utilization. Currently, a common heuristic strategy used to choose antibodies for their research relies on the number, or perceived quality, of citations in the published literature. This may result in the ongoing use of poorly performing antibodies, especially if they have already been used extensively in the published literature, or if they have been used in particularly influential papers. This creates a vicious cycle, that the current research environment does not have effective mechanisms to stop.

We believe that multiple stakeholders need to be engaged in the process of changing practices and behavioral norms that have led to this situation. Psychological frameworks such as the COM-B model^16^ suggest a number of key dimensions that determine behavior and influence the success of behavior change interventions. These typically include individual capability and motivation for certain behaviors, as well as the environment and opportunity that is afforded to them. In the context of antibody choices, individual scientists require knowledge to make informed antibody choices, adequate time, and resources to perform validation and reliability experiments, awareness about the consequences of insufficient antibody validation, and higher recognition and better evaluation of their research.

**Figure 2.**
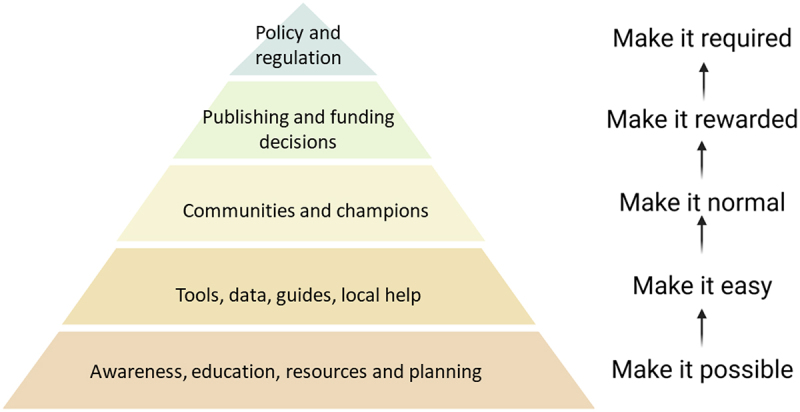
A strategy for improving the use of antibodies in research.

Behavioral and cultural change is notoriously difficult, and likely represents an important barrier to progress. Again, behavior change models (e.g., the Behavior Change Wheel) from the field of psychology might help to identify appropriate interventions.^16^ Stakeholders like funders and publishers can play key roles because they determine the research environment and help to shape incentives for better practice by individual scientists. However, any such action will likely require consensus and co-ordinated, global action between multiple stakeholders, each with its own priorities and limitations. We believe that an approach based on an understanding of end-user behavior and the Centre for Open Science strategy for research culture change is necessary to start to define interventions that will improve research that uses antibodies (Figure 2). In this context culture refers to behaviors of the community that represent the norm.

Summary of potential areas for consideration for stakeholders to develop action plans toward making best practices more feasible, easy, and rewarding.

### Technical solutions

Several efforts have made significant progress in validating antibodies at scale, either as a single initiative, or to collate data derived from different sources. A 2008 publication linked with the Human Protein Atlas project indicated that more than half of some 20,000 commercial antibodies were not suitable for immunohistochemistry.^17^ Their large-scale validation effort primarily evaluated the consistency of antibody staining with bioinformatic data, which the authors acknowledge required subjective comparisons between data generated using antibodies to bioinformatic prediction methods. The Human Protein Atlas represents a resource used by scientists to understand the relationship between antibody staining and RNA expression, or between two independent antibodies targeting different epitopes of the same protein. These methods do not always exclude the possibility of poor antibody selectivity for the target.

Another important community-based antibody characterization initiative is the Human Cell Differentiation Molecules (HCDM) organization that runs the Human Leukocyte Differentiation Antigens workshop.^18^ The organization has operated since 1982 and has held ten workshops that have resulted in the naming of over 350 “Cluster of Differentiation” or CD markers that are widely adopted by the immunology community. Each marker represents a human leukocyte surface antigen. The characterization of these markers relies on a community of immunologists sharing monoclonal antibodies directed at human leukocyte surface antigens in a blinded manner and sharing the staining pattern of leukocytes by multicolor flow cytometry at each workshop. The focus of the organization to date has been human leukocyte surface antigens, using these to define robust and reproducible leukocyte populations for study using flow cytometry. In relation to the 5 pillars approach described above this would constitute orthogonal validation, and in some instances independent antibody validation, when multiple independent clones are available.

YCharOS is another collaborative initiative that aims to characterize antibodies using genetic strategies for the entire human proteome, in collaboration with primary antibody manufacturers.^14,19,20^ They have evaluated approximately 1000 antibodies directed at around 100 targets to date, relying heavily on the use of CRISPR-Cas 9 knockout lines for use as isogenic controls in western blotting, immunofluorescence, and immunoprecipitation. They present open head-to-head comparisons of multiple commercial antibodies, using knock-out cell lines compared to isogenic wild-type controls, using standardized protocols in three different applications. The open data presented shows a quality control pass rate of 49.8% for western blot, 43.6% for immunoprecipitation and 36.5% for immunofluorescent (IF) staining in an analysis of the first 614 antibodies characterized.^8^ Under the conditions used, more than half of antibodies were unable to selectively label, for western blotting and IF, or efficiently precipitate the target of interest (see 8).

Their work also suggested that orthogonal controls, where antibody staining is compared to RNA expression for example, may not be a reliable indicator of selectivity, as evaluated using the more robust genetic knockout; particularly in immunofluorescence.^14^ They identified large numbers of poorly performing antibodies that have been used in the literature, and when used for IF (the worst-performing application), were presented without any validation data 87.5% of the time. An important finding was that, within the sample they studied, recombinant antibodies performed better for all three applications within their pipeline than hybridoma-derived monoclonal antibodies and animal-derived polyclonal antibodies. One of the potential reasons for this is that there can be substantial lot-to-lot variation between polyclonal antibodies, and that validation of every lot may not be feasible for manufacturers or end users.

Importantly, they also found selective antibodies among those tested and provided by commercial sources for most of the proteins they studied. Their work so far has resulted in companies altering the recommended usages, or removing from catalogs, over 200 antibodies. This is a particularly important contribution of the vendors and YCharOS, eliminating the use of poorly selective antibodies at source and providing more reliable usage recommendations. Their data highlights the value to researchers of identifying good performing antibodies in the sea of those available, the importance of making such data publicly available, and the contributions vendors are making in improving this situation.

As YCharOS characterizes the antibodies under one (or two) specific set(s) of conditions, researchers need to be aware that antibody performance may vary between cell type or experimental conditions. YCharOS recommends that end users perform at least siRNA/shRNA knockdown controls in their relevant system to further confirm selectivity under the specific experimental conditions (including reagents and protocols) and in the sample types of interest, which may differ from those used in the YCharOS characterization pipeline.^20^

Over six million antibodies are identifiable in the biomedical research literature,^21^ altogether directed at over 30,000 proteins from multiple species and modified proteins (e.g., post-translational modifications and genetically engineered varieties of fluorescent proteins, small tags). Thus, despite impressive progress by YCharOS, much further characterization work is needed. This will require substantial increases in resources and mechanisms to maximize the impact of available characterization data.^1^

### Open quality control data sharing

Another determinant of end-user behavior is open data sharing because it has the potential to improve the standards of research (and reduce waste) by allowing researchers to identify antibodies that are more likely to be suitable for their application. Again, open quality control data interacts with psychosocial determinants of behavior because it enables users to make more informed choices about antibodies.

There is an abundance of databases and data tools that may help scientists make better antibody purchasing decisions. Yet our focus group data suggests that users are unaware of them or do not know how to use them. Previous authors have described the databases in a more systematic way,^22^ although a formal comparison of their ability to identify better-performing antibodies has not been presented. To address this shortfall, we are currently systematically evaluating the performance of the data tools independently, and in future will look to produce guidance on how best to use them to reduce the risk of poor antibody performance. Below we describe our opinion of the relative merits and pitfalls of some key databases.

Data from the proteins to date characterized by YCharOS is rapidly disseminated throughout the research community via F1000, Zenodo, and the RRID portal. A separate F1000 Antibody Validations gateway also contains 16 articles with data supporting the specificity of some antibodies. This gateway also contains several reviews about antibody validation. The RRID portal allows users to search for antibodies, filtering by whether users (or organizations such as YCharOS) have submitted validation data. The RRID initiative aims to improve research reproducibility by ensuring that all research resources are clearly and unambiguously identifiable. The reporting of research antibody use in the literature has also been shown to be problematic, with an analysis from 2013 showing high frequency of papers not reporting sufficient details to enable identification of which antibody had been used.^23^ The use of RRIDs has been associated with an improvement in the reporting standards where their use has been encouraged by journals.^24^

In the case of antibodies, the unique identification of the different reagents is a key challenge, an issue further compounded by the fact that the same antibodies are being sold by multiple vendors with a lack of transparency.^13^ Users can upload data themselves via Biomed Resource Watch. This provides one mechanism to enable the dissemination of data related to antibody performance. An advantage of Biomed Resource Watch is that it also allows for the submission of data that suggests concerns about antibody specificity (rather than only positive data). In our experience however, to date there is a paucity of user-generated or published data regarding antibody specificity linked to RRIDs.

Several other databases and mechanisms also allow data or user reviews to be associated with antibodies. Some antibody vendor websites allow users to submit open reviews. Biocompare has an antibody search engine that allows users to quickly find vendor data, images from the literature and user reviews submitted by scientists. For now, there does not appear to be a way to rapidly find antibodies that have validation data, although it is possible to quickly find antibodies that have user generated reviews.

Independent antibody user reviews are also hosted by pAbmAbs, a website with no commercial interest in antibody manufacturing or selling antibodies. Reviews can include links to literature; reviewers are also able to submit images. There are 902 proteins listed in their index, but only some of these include antibodies that have been reviewed. We have not yet established the proportion of these that share, or link to, robust data.

Labome performs ongoing manual curation of the published literature to identify studies that include antibody validation.^25^ This has, to date, identified 1445 studies in the literature that show knockout cell line/animal data that support the use of a specific antibody. Their database includes images from the publication, allows users to assess the quality of validation data more rapidly, and links to the primary publication. Partial access to the database can be purchased, and validated antibody reviews can be accessed for free.

Their database does include multiple examples of knockout or knockdown validation for the same protein from the literature. The inclusion of multiple studies of the same antibody or protein (with multiple applications) can be useful for antibody purchasers. It should be noted that the website also provides commercial listings for many antibodies that do not have supporting data, and care needs to be taken to understand which antibodies are within the validated antibody database and which are simply commercial product listings. We recommend accessing the published data directly prior to purchasing any antibody.

Antibodypedia^4^ lists antibodies from 101 providers and allows antibodies to be filtered according to validation data. There are six categories of validation data provided: genetic, orthogonal, independent antibody, recombinant expression, capture mass spectroscopy, and other. These represent the five pillars of validation as described by Uhlen et al.^6^ and an additional category (other). In our experience with a small number of target proteins, many targets have only “other” validation data. Like the other data tools described, it also has the potential to allow users to find antibodies with robust validation data. It also allows for the submission of user-generated data, but currently most data appear to be from suppliers. It also links antibodies to their unique RRID in The Antibody Registry in some instances.

Other tools include CiteAb,^26^ which ranks antibodies by the number of times they have been used in the literature. We find this a useful tool for finding papers that use given antibodies. It also has features that may help users find validation data from the literature more efficiently. However, we have found multiple examples of highly used antibodies in the literature with performance issues, and examples of lesser-used antibodies with better performance (e.g., TRPA1,^9,10^ C9ORF72^27^). Many of the antibodies with poor performance in all tested applications in the YCharOS workflow have also been cited many times.^14^ Citation count should not be used as an indicator of antibody selectivity for this reason. We believe that the images in the literature are likely affected by publication bias, where data supporting an antibody’s performance will be overrepresented, relative to data that suggests issues with antibody selectivity. Citations will also tend to be high on antibodies that were first to market, which will frequently be older polyclonal antibodies, rather renewable antibodies (see above).

Other initiatives include The ABCD database, a project to uniquely identify recombinant antibodies by their sequence data. It is supported by a publication platform called antibody reports for recombinant antibody validation data, which now contains 14 issues, each of which shows validation data for around ten antibodies. Recombinant antibodies have advantages in terms of potentially having a limitless supply, minimizing variations from lot-to-lot, and more ethical production in terms of reduced use of animals.

Benchsci^28^ is an artificial intelligence tool that allows users to identify images from the literature rapidly. It incorporates the ability to filter images, including antibody data by application of interest, reactivity, tissue of interest and other categories. It also includes the ability to filter by knockout/knockdown, which in our experience does bring up relevant publications from the literature. This tool facilitates the search of the literature for relevant data about antibodies. We have not yet seen an assessment of its precision. At this point it appears that this data needs human evaluation and checking, and formal evaluation of the tool’s performance in specific tasks would help inform the scientific community of its strengths and weaknesses.

In our opinion the ideal tool(s) would help uniquely identify every antibody (or alternative affinity or binding reagent), link this to high-quality characterization data, and clearly identify antibodies with potential issues, linking them to every publication they are used in. The tool would be embedded within the workflow of research funders, ethics review committees and publishers, thereby allowing them to rapidly change their practices as new data becomes available. We advocate for publications that have used nonselective antibodies without robust validation data to be flagged, so that readers are aware of important caveats in the reliability of the data.

There are many challenges in making this data tool a reality. For now, some degree of (expensive and difficult) manual data curation is likely to be necessary, given the complex nature of the underlying data and methods. In addition, such data-sharing tools are limited by the willingness of the scientific community to engage with these data-sharing and curation exercises. Scientists are time-poor, and meaningful incentives and/or data-sharing mandates may be needed.

### The only good antibodies community and partners

Our progress review above has showcased ongoing efforts in the improvement of technology and data-sharing solutions, but highlighted the need for a better understanding of psychosocial factors that determine end-user behavior. The problem of poorly performing antibodies is multifactorial and requires collaboration and cross-disciplinary work to solve. For this reason, we have created the Only Good Antibodies (OGA) community, a diverse international collaboration of basic, clinical, data and behavioral scientists and technicians from academia, industry, and nonprofit organizations. The OGA community aims to work with all stakeholders to accelerate fundamental and translational science by offering a holistic behavioral perspective on how to increase the availability and use of selective antibodies and eliminate the use of poorly performing antibodies.

**Figure 3.**
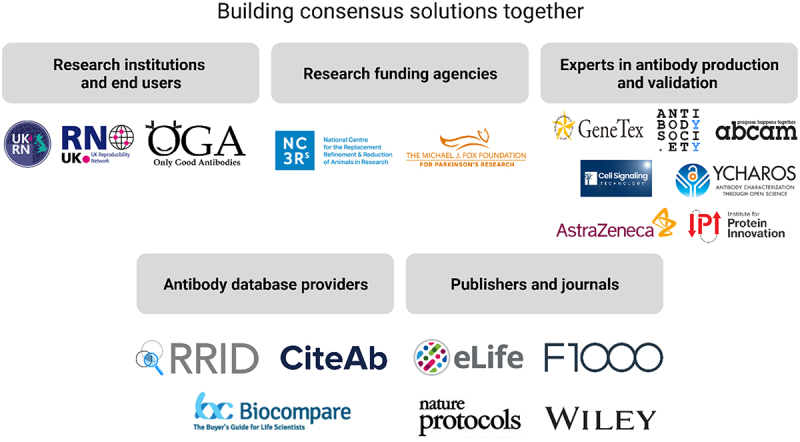
Working with stakeholders across the scientific community to develop consensus solutions.

The OGA community is actively engaging various stakeholders across science; our current partners include those mentioned in this opinion article such as YCharOS and RRID. We also work with The Antibody Society to disseminate educational resources, empower researchers to choose better antibodies and perform better validation, and to advocate for necessary standards and policies. Further organizations have formalized their support for this initiative, and we welcome new stakeholders (Figure 3).

Research institutions and end users: UK Reproducibility Network (UKRN) and the Only Good Antibodies community (OGA); research funding agencies: National Centre for the Replacement, Refinement and Reduction of Animals in Research and the Michael J Fox Foundation for Parkinson’s Research; experts in antibody production and validation: Genetex, the Antibody Society, Abcam, Cell Signalling Technology, YCharOS (Antibody Characterization through Open Science), AstraZeneca, and the Institute for Protein Innovation; antibody database providers: the RRID initiative, CiteAb and Biocompare; publishers and journals: eLife, F1000, Nature protocols and Wiley.

## How you can help

The aim of the OGA is to raise awareness of the problem of poorly performing antibodies and develop evidence-based interventions, frameworks, and policies – and evaluate their impact. To do this, we need to engage with all stakeholders with your help. For more specific information on how this issue might affect you, and how you can get involved, please see below.

### Medical charities and research funders

We want to find the best way to help research funders to support projects that are more likely to succeed by ensuring they use selective antibodies, and that antibody information forms part of their assessment of research projects. We are looking for partners to collaboratively develop processes that work both for the funder and scientists that address this issue, and thereby result in more successful projects and treatments.

### Academic publishers and journal editors

We wish to increase the impact published articles have on the biological and medical sciences community. We can do this by helping to collaboratively develop tools/frameworks for research assessment and reporting standards. These can be used with the community of peer reviewers/scientists and will enable you to screen out low-quality research (possibly at an earlier stage of review) and allow you to focus on transformative research. We seek to collaborate with you to develop feasible and effective ways of working that meet the needs of all stakeholders.

### Ethics committees, regulatory authorities, and institutions

We believe that review of research ethics and the regulatory requirements of animal, clinical and human tissue research represent opportunities to address this problem. We want to work with you to develop frameworks to assess research projects that meet the needs of the relevant committees, authorities and needs of the wider research community.

### Antibody users/researchers

As the end users of antibodies, we believe you are some of the early beneficiaries of the OGA community and forum. We invite you to formally join our community and support us by contributing to our focus groups and surveys, to help us understand your views and concerns, and to give us ideas about how to eliminate this problem. We also understand that this issue can have substantial negative consequences for your career. Our forum provides a place to share your problems and concerns about this issue, and to find support from your peer community.

### Antibody manufacturers and vendors

You provide a vital service in science by producing and distributing these important reagents. Improving the quality of your products and the details of any data used to demonstrate antibody utility will allow you to increase your sales and charge fair prices for better antibodies. This will improve your reputation and potentially your market share. If you do not already work with YCharOS, please contact them: https://ycharos.com/contact/. We are also looking for partners to improve the information available to antibody users at the point of sale and metrics we can use to track sales and citation data for well-characterized antibodies.

### Media and campaigners

We believe this issue is of high importance to the public because of the impact it has on the use of taxpayer money, animal use, and samples donated by patients and research participants. To eliminate this problem, help us reach a wider audience to make the case for its importance by publishing on the issues and progress toward solutions.

To share your insight on the antibody reproducibility crisis or provide feedback to the OGA community, please contact the corresponding author.
